# Sclerotherapy for Aneurysmal Bone Cyst: A Single-Center Experience

**DOI:** 10.7759/cureus.18469

**Published:** 2021-10-04

**Authors:** Deepak Kumar, Sanjeev Kumar, Dharmendra Kumar, Brij Mohan Patel, Ashish Kumar, Santosh Kumar, Shah Waliullah

**Affiliations:** 1 Orthopedic Surgery, King Georges Medical University, Lucknow, IND; 2 Department of Orthopedics, Mahamaya Government Medical College, Ambedkar Nagar, IND

**Keywords:** complications, clinical efficacy, polidocanol 3%, sclerotherapy, aneurysmal bone cyst

## Abstract

Introduction

Sclerotherapy offers an alternative to surgery to treat an aneurysmal bone cyst (ABC). The present study's main objective was to assess the radiological efficacy of sclerotherapy in the healing of the cyst cavity secondary to biopsy-proven ABC on X-rays and assess clinical efficacy on pain, recurrence, and complications.

Materials and methods

Between 2016 and 2018, 26 patients (12 females, 14 males) with biopsy-proven ABC treated by sclerotherapy were included. All patients received an injection of polidocanol 3% intralesional as standard treatment under fluoroscopic guidance. Ossification was assessed on plain X-ray, and the pain was evaluated on a visual analog scale (VAS).

Results

Ossification was complete in 24 (92.3%) patients and partial in two (7.7%) patients. Eighteen patients (70%) were pain-free at the end of three months. There was an improvement in the VAS score, and clinically, there was a significant reduction in pain and swelling. Two patients developed recurrence within two years of follow-up, treated successfully by the re-application of intralesional polidocanol 3% injection.

Discussion

Sclerotherapy provides an effective, minimally invasive treatment for ABC and is particularly useful for deep lesions, challenging access for surgery and potentially damaging vital structures. The use of percutaneous polidocanol 3% under fluoroscopic control seems to improve the risk/benefit ratio. Its clinical and radiological efficacy makes sclerotherapy an alternative treatment option in ABC.

Level of evidence

IV, prospective study

## Introduction

An aneurysmal bone cyst (ABC) is a benign bone tumor with lots of controversy regarding its origin and management. In 1942, Jaffe and Lichtenstein first described aneurysmal bone cysts (ABCs) [1]. As per World Health Organization (WHO), ABC was defined as ''a benign cystic lesion of bone composed of blood-filled spaces separated by connective tissue septa containing fibroblasts, osteoclast-type giant cells and reactive woven bone'' [2-3]. Typically, this tumor occurs in the first two decades but a few cases are diagnosed later in life. ABC occurs equally in males and females means there is no sex predilection. ABC's prevalence is 1.4 cases per 100,000 individuals, constituting about ∼1% of all bone tumors. An ABC is a benign tumor, highly vascular, and osteolytic lesion of unknown etiology [4-5]. It is an expansile osteolytic tumor-like lesion with multiple blood-filled cavities [6]. It can affect any bone in the body, but most commonly in the long bones, especially in the eccentric metaphysis of the femur and tibia [7].

An ABC may be of two types based on origin. It may be primary or arise secondarily in other benign or malignant bone tumors [2-3]. Oliveira et al. differentiated primary and secondary ABC cytogenetically as primary ABC exhibits USP6 or CDH11 rearrangements in their cells in about two-thirds of the cases [8]. Clinically, ABC may present as a latent form or an active form and rarely as an aggressive form [9]. The clinical presentation is mainly pain, swelling, and possible deformity due to its ability to expand, leading to pathological fracture and movement restriction depending upon the tumor's site and size [10].

Many treatment options are available; traditionally, they were treated by curettage, with and without bone grafting [11-12].ABC can also be treated by selective arterial embolization or radiotherapy, or a combination of both [13-14]. Other methods include direct injection of the cyst with a sclerosing agent such as polidocanol 3% [15-16]. For ABC, Polidocanol was documented by Jain et al. [15] and used effectively for ABC. Percutaneous sclerotherapy is a safe alternative to surgery for ABC, associated with lesser morbidity. Here, we report our experience of percutaneous sclerotherapy for skeletal ABC. We prospectively evaluated the radiological and clinical outcomes of the skeletal ABC patients treated with percutaneous intralesional polidocanol 3% injection.

## Materials and methods

The study was approved by our institutional ethics committee (IRB no: 89 ECM II B IMR-F/P4). A single-centric, prospective cohort study was carried out at our institution in the department of orthopedic surgery between 2016 to 2018. The study included 26 biopsy-proven patients of primary ABC. Patients presented with radiological features of ABC were taken and underwent a biopsy. Only after histopathological confirmation of primary ABC, they were recruited in the study. The patient and their parents were explained the pros and cons of the percutaneous sclerotherapy and curettage and bone grafting procedure to treat ABC. Patients willing for sclerotherapy procedures with consent were taken for the study. Any patients who were operated on previously for the treatment of ABC or taken treatment of skeletal ABC elsewhere or suffering from any other tumor condition were excluded from the study. All patients were managed by either fluoroscopic or CT-guided sclerotherapy with intralesional inj polidocanol 3% (Hydroxypolyaethoxydodecan, Samarth Life Sciences, Solan, India). A single vial of polidocanol injection was available as 2 ml ampoules and each ml contains 30 mg of polidocanol. Nine lesions were located in the upper limb; 15 in the lower limb and two in the pelvis (Table 1). Preoperative evaluation included clinical, radiological, and histopathological assessment.

**Table 1:** Demographic and radiological characteristics

Patient	Age at presentation (in years)	Gender	Location	Presentation	Size of lesion on X-ray in cm	No. of injections	Degree of ossification	Duration of follow-up (in months)
1.	15	F	Distal 1/3^rd^ Clavicle	Pain & Swelling	4X3	5	Complete	24
2.	9	M	Distal 1/3^rd^ shaft Ulna	Swelling & Pain	2X2	3	Complete	36
3.	12	F	2^nd^ Metatarsal	Pain	1X1	2	Complete	28
4.	8	F	Proximal Humerus	Swelling and pain	3X2	3	Incomplete	24
5.	13	F	Iliac Wing	Pain	3X3	4	Complete	34
6.	14	M	Proximal tibia	Swelling and pain	3X2	4	Complete	40
7	12	M	Distal tibia	Pain and swelling	3X2	3	Incomplete	45
8.	12	M	Proximal femur	Pain & Swelling	2X2	3	Complete	48
9	11	F	Humeral head	Swelling	2X1	3	Complete	42
10	14	F	Ilium	Pain	2X3	4	Complete	44
11	8	M	Distal tibia	pain	2X2	3	Complete	47
12	6	F	Proximal humerus	Pain and swelling	1X2	2	Complete	30
13	8	M	Proximal humerus	Pain and swelling	3X4	4	Complete	43
14	12	M	Distal ulna	Pain and swelling	2X2	2	Complete	34
15	14	F	Distal tibia	pain	4X3	4	Complete	32
16	12	F	Second metatarsal	Pain and swelling	1X2	1	Complete	25
17	14	M	Proximal humerus	Pain and swelling	4X2	3	Complete	28
18	12	M	Proximal femur	pain	4X3	2	Complete	29
19	14	M	Proximal femur	Pain and swelling	4X4	3	Complete	41
20	8	F	Distal tibia	Pain and swelling	2X2	3	Complete	43
21	6	F	Proximal femur	Pain	2X2	2	Complete	28
22	10	M	Distal fibula	Pain and swelling	1X1	1	Complete	41
23	11	M	Proximal tibia	Pain and swelling	2X1	4	Complete	31
24	14	F	Distal radius	Pain and swelling	2X1	1	Complete	26
25	12	M	Fibular head	Swelling	1X1	1	Complete	25
26	14	F	Calcaneum	Swelling and pain	1X1	2	Complete	36

For the radiological evaluation, radiographs in both anteroposterior and lateral views were taken. All patients underwent magnetic resonance imaging of the affected part. In addition, computed tomography (CT) was done in selective cases to assess bony anatomy and cortical breach. For making the histological diagnosis, either percutaneous trephine biopsy or open biopsy was done. The cyst volume was calculated by multiplying the length, width, and depth of the cyst. The length and width of the cyst were measured by taking the maximum dimension on the anteroposterior view, and the depth was measured on the lateral view with its maximum extent [16]. After biopsy and histopathological confirmation, patients with cortical breach were initially allowed for three months, for the cavity to be get healed. Treatment was undertaken under general anesthesia or regional anesthesia depending upon the age of the patient and site of the lesion. The needle was placed into the lesion under fluoroscopy or CT guidance. Patients and their families were explained the advantages and risks of the procedure, complications, and future surgery requirements, and then written informed consent was obtained.

Procedure

Polidocanol 3% was injected into the lesion under fluoroscopic or CT guidance using an 18G needle. After needle placement, fluid was aspirated from the lesion, and the presence of fluid suggested active disease. After fluid aspiration, 3% polidocanol was gradually injected into the lesion. For every cubic cm volume of the lesion cavity, about 1 ml of 3% polidocanol was injected. The maximum amount of sclerosant was not allowed more than 10 ml, irrespective of the size of the lesion, to avoid adverse reactions due to sclerosant [16]. Patients with suspected breach were given a small amount of drug initially at a first dose and very slowly under fluoroscopic guidance. As the cavity showed sclerosis and healing in follow-up, a larger amount of drug volume was injected as required.

At each follow-up visit, the patients were evaluated clinically and radiologically. The follow-up was done at six weeks, 12 weeks, and then at every three-monthly intervals. The endpoint of the treatment was defined clinically as the resolution of pain and radiologically as the evidence of reformation of cortical wall thickness and the cessation of growth of the lesion. Radiological evaluation at follow-up was done by radiographs only. The first three-month follow-up was taken as the first endpoint. Pain evaluation was done by VAS score; the radiological assessment was done for ossification (partial or complete) and tumor size change.

Further repetition of sclerosing agent depended upon the achievement of the clinical and radiological endpoints at the second follow-up visit (three months). After three months of follow-up, if the patient had persistent pain or radiologically, there was an increase in tumor size or absence of healing, the injection was repeated. At each follow-up visit, patients were evaluated clinically and radiologically. Clinically, patients were assessed for the presence of pain or any complication related to the treatment, and radiologically, patients were graded as per grading given by Rastogi et al. [16].

Statistical analysis

Data were analyzed by SPSS software (SPSS Inc., Chicago, IL, USA) for Windows program (15.0 version). The continuous variables were measured by mean (standard deviation) or range value as applicable. The dichotomous variables were presented in number/frequency and analyzed using the chi-square test or Fisher's exact test. For comparison of the means between the two groups, analysis by student's t-test was used. With a 95% confidence interval, a p-value of 0.001 was considered significant.

## Results

This study included 26 patients, of which 14 were females and 12 were males, with a mean age of 11.35 years (6 to 15 years). The mean follow-up period was 34.77 months (24 to 48). The mean number of injections given per patient was 2.46 (1 to 5). Four cases required only one injection for treatment (Table 1). There were two cases lost to follow-up.

**Figure 1:**
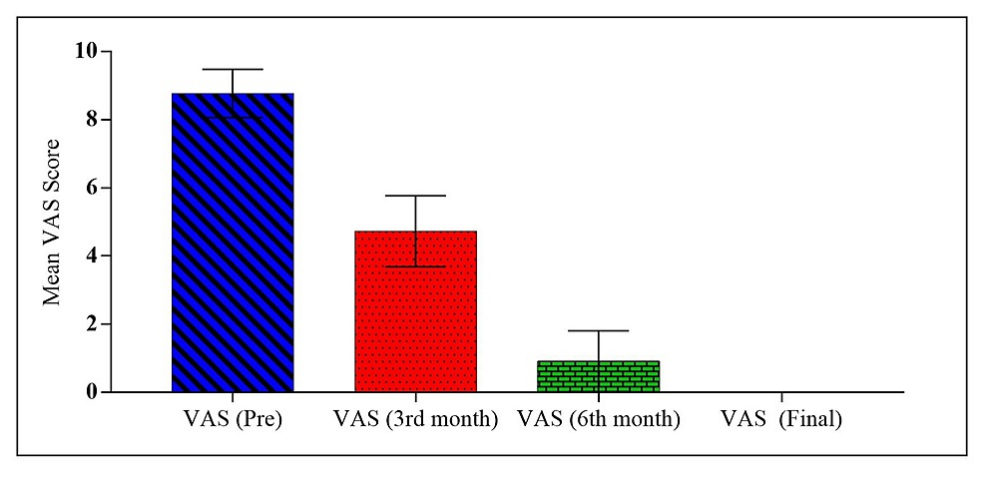
Comparison of VAS score at different intervals VAS: visual analog scale

The mean pre-injection VAS score was 8.77, and the post-injection score improved to 1.46 at the two years follow-up, which was significant (Table 2, Figure 1).

**Table 2:** Comparison of VAS score at different follow-ups VAS: visual analog scale; ANOVA: analysis of variance

	VAS score (Pre)	VAS score (3^rd^ Month)	VAS score (6^th^ Month)	VAS score (2 years)	p-value
Mean ± SD	8.769 ±0.710	4.731 ± 1.041	0.9231 ± 0.891	0 ± 0	ANOVA: F=700.1 p<0.0001*
Minimum	7	3	0	0	
25% Percentile	8	4	0	0	
Median	9	5	1	0	
75% Percentile	9	6	2	0	
Maximum	10	6	3	0	

**Figure 2:**
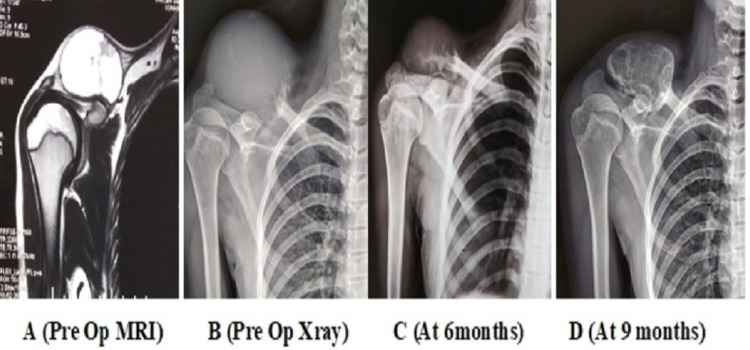
Depicting ossification of the lateral clavicle ABC mass after sclerotherapy ABC: aneurysmal bone cyst

**Figure 3:**
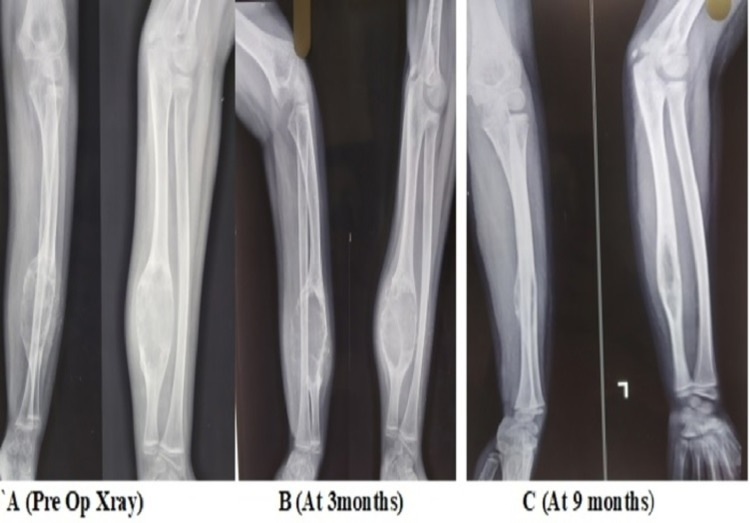
Ossification of a distal 1/3rd ulnar shaft ABC tumor ABC: aneurysmal bone cyst

**Figure 4:**
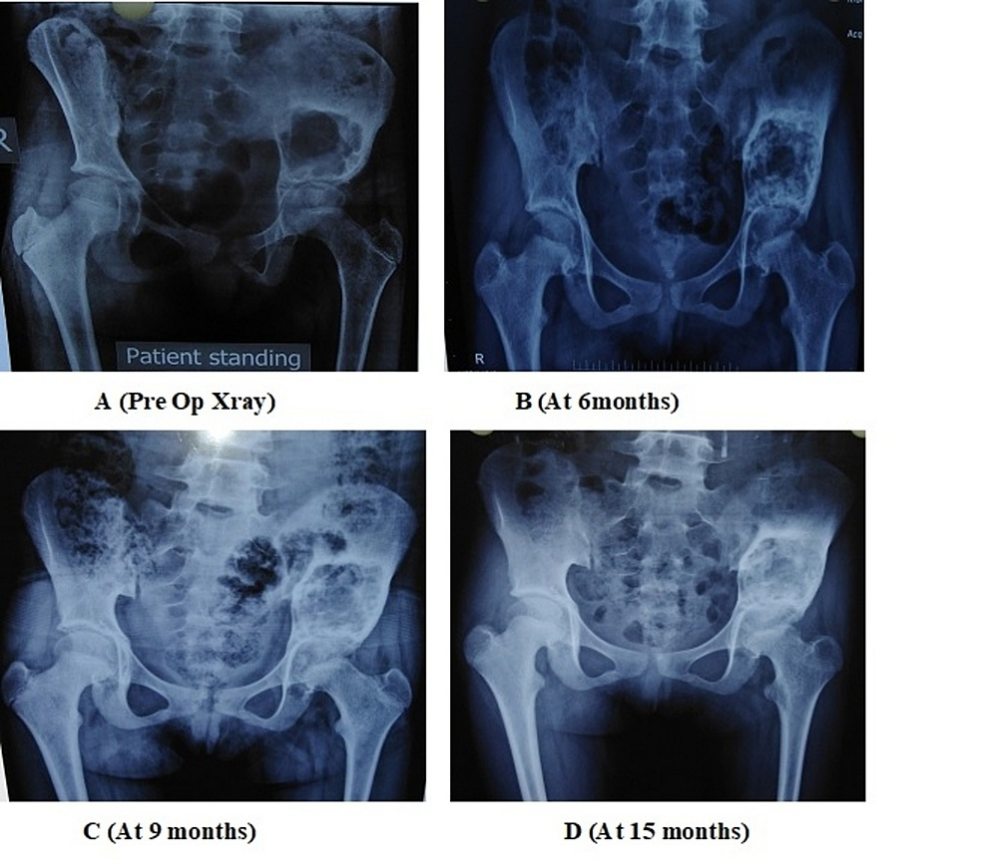
Depicting the ossification of the cystic cavity in a 13-year-old child with ABC of the iliac wing ABC: aneurysmal bone cyst

There was no residual pain following injections in 24 patients, with satisfactory radiological healing (Figures 2-4).

In a follow-up period of two years, 22 patients were in Grade I while four patients were in Grade II (Table 3) as per grading recommended by Rastogi et al. [16]. Two patients showed recurrence; the first patient reported recurrence after 12 months while the second reported at 14 months of therapy with pain and an increase in the lesion size radiologically. These patients were managed by repeat sclerotherapy and healed uneventfully at a further six months of follow-up.

**Table 3:** Radiological healing at different follow-up

N=26	At three months	At six months	At 2 years	At final follow-up	P-value
Grade I	4	10	22	21	ꭓ=40.46 p<0.0001*
Grade II	18	12	4	3	
Grade III	2	3	0	0	
Grade IV	2	1	0	0	

Complications

We observed local complications at the site of injection. Five patients developed skin induration at the injection site, and two patients had skin hypopigmentation following injection. There was no case of anaphylaxis, infection, or any significant adverse reactions.

## Discussion

ABC is a bone tumor with controversial treatment. There are various options for the treatment of ABC, which includes curettage with or without bone grafting, complete excision or subtotal excision with or without reconstruction, arterial embolization, bone marrow injections, intralesional drug injections (steroid, calcitonin, or doxycycline), radiation therapy, denosumab injection, and sclerotherapy [14-24]. Sometimes, lesions heal spontaneously after biopsy and pathological fracture [25].

Sclerosants have the irritant property, cause direct damage to the inner endothelial lining of the blood vessel, and result in thrombotic occlusion of blood vessels [16]. Polidocanol, a sclerosant, has been used safely to treat varicose veins, oesophageal bleeding due to varices, intestinal vascular malformations, telangiectases, and venous malformations of the head, neck, and limbs [16,26-27]. Polidocanol has also been used as a local anesthetic and antipruritic agent in ointment form [28]. Polidocanol has been used safely to treat ABC of the skeletal system without any severe side effects. However, Gupta et al. reported a life-threatening adverse event with an intralesional application of polidocanol in managing recurrent ABC of the proximal femur in a three-year-old child, so it is recommended to perform sclerotherapy procedure in the operation theater with proper anesthesia support so as to tackle any adverse reaction [28].

Rastogi et al., in their study on 72 patients with ABC, reported a 97% success rate in treatment with sclerotherapy [16]. He observed a 75% clinical response at the end of treatment of the mean 11.4 months, and this response improved further at the final follow-up of a mean of 34 months. Otte et al. reported an efficacy of 97% in a series of 38 patients with ABC treated with sequential sclerotherapy [29]. In our study, we observed a comparable 92.3% healing of lesions in our patients.

In a recent study, Deventer et al. reported that out of 32 ABC patients treated with polidocanol sclerotherapy, 90.6% of patients had the persistent disease and 34.5% of patients further required treatment [30]. We feel the probable reason for these less satisfactory results is a radiological evaluation with an MRI scan. He took an MRI scan to assess regression in the volume of the cyst during follow-up. MRI is a sensitive and better evaluation modality to measure cyst volume; it shows persistent fluid levels during follow-up, even after the pain resolves. We observed that patients reported resolution of pain earlier. The majority of patients were pain-free at the end of the first clinical endpoint at three months; however, radiological improvement lags behind clinical improvement. Radiological healing in the form of sclerosis and cortical wall thickening will be better evaluated by CT scan than MRI and conventional radiographs. We propose future studies with CT as a follow-up evaluation modality that will give a better insight into access radiological healing.

Our study noted recurrence in two patients, and both occurred within two years of the treatment. Both were successfully treated by further sclerotherapy. Rastogi et al. [16] reported recurrence in two patients (2.8%) while Puri et al. [11] observed recurrences in four patients and all after two years in his series. The majority of studies observed recurrences within two years of follow-up [12,14,16]. We also observed recurrence in two years in our patients so we recommend that skeletal ABC patients treated with sequential sclerotherapy be followed for a minimum of two years.

Our study has certain limitations. Due to the single-centric, prospective analysis, results cannot be generalized. We have not compared sclerotherapy with surgery and other treatment modalities. Multicentric, randomized control studies can provide further robust evidence to prove the usefulness of sclerotherapy as compared to other modalities for the treatment of skeletal ABC.

## Conclusions

This study shows that percutaneous sclerotherapy is an effective, simple, minimally invasive treatment for ABC and is particularly useful for deep lesions, challenging access for surgery and potentially damaging vital structures. The use of percutaneous polidocanol 3% under fluoroscopic or CT control seems to improve the risk/benefit ratio for skeletal ABC patients. With further future randomized studies, sclerotherapy's clinical and radiological efficacy can make it a viable and acceptable alternative to conventional surgical techniques.
